# Improving Biocompatibility of Polyester Fabrics through Polyurethane/Gelatin Complex Coating for Potential Vascular Application

**DOI:** 10.3390/polym14050989

**Published:** 2022-02-28

**Authors:** Wei Wang, Ziyi Zhou, Na Liu, Xiaopei Zhang, Hua Zhou, Yuanfei Wang, Kuanjun Fang, Tong Wu

**Affiliations:** 1 College of Textile & Clothing, Qingdao University, 308 Ningxia Road, Qingdao 266071, China; wangviyeah@163.com (W.W.); zhouhua3216@163.com (H.Z.); 2 Collaborative Innovation Center for Eco-Textiles of Shandong Province and the Ministry of Education, 308 Ningxia Road, Qingdao 266071, China; 3 State Key Laboratory for Biofibers and Eco-Textiles, 308 Ningxia Road, Qingdao 266071, China; 4 Collaborative Innovation Center for Eco-Textiles of Shandong Province, 308 Ningxia Road, Qingdao 266071, China; 5 Institute of Neuroregeneration and Neurorehabilitation, Qingdao Medical College, Qingdao University, Qingdao 266071, China; zzy10162022@163.com (Z.Z.); A1349747636@163.com (N.L.); xp_dreamfly@163.com (X.Z.); 6 Department of Cosmetic and Plastic Surgery, Affiliated Hospital of Qingdao University, Qingdao 266071, China; 7 Central Laboratory, Qingdao Stomatological Hospital Affiliated to Qingdao University, Qingdao 266001, China

**Keywords:** polyurethane, gelatin, coating, fabric, biocompatibility

## Abstract

Medical apparatus and instruments, such as vascular grafts, are first exposed to blood when they are implanted. Therefore, blood compatibility is considered to be the critical issue when constructing a vascular graft. In this regard, the coating method is verified to be an effective and simple approach to improve the blood compatibility as well as prevent the grafts from blood leakage. In this study, polyester fabric is chosen as the substrate to provide excellent mechanical properties while a coating layer of polyurethane is introduced to prevent the blood leakage. Furthermore, gelatin is coated on the substrate to mimic the native extracellular matrix together with the improvement of biocompatibility. XPS and FTIR analysis are performed for elemental and group analysis to determine the successful coating of polyurethane and gelatin on the polyester fabrics. In terms of blood compatibility, hemolysis and platelet adhesion are measured to investigate the anticoagulation performance. In vitro cell experiments also indicate that endothelial cells show good proliferation and morphology on the polyester fabric modified with such coating layers. Taken together, such polyester fabric coated with polyurethane and gelatin layers would have a promising potential in constructing vascular grafts with expected blood compatibility and biocompatibility without destroying the basic mechanical requirements for vascular applications.

## 1. Introduction

Cardiovascular diseases are known for their high incidence rate and mortality around the world. According to statistics, the number of people dying of cardiovascular diseases exceeds 15.2 million every year and the number is yearly increasing [1]. The medical implants (artificial blood vessels, artificial heart valves, and cardiovascular stents) usually play a critical role in the treatment of cardiovascular diseases [2]. However, the implants are first exposed to blood after implantation, which will lead to blood compatibility problems [3]. The existence of implants usually causes considerable side effects—such as coagulation, intimal hyperplasia, infection, and thrombosis—which lead to the failure of implantation [4,5,6,7]. In previous studies, anticoagulant drugs such as heparin were added to the implants to avoid coagulation reaction. For example, heparin and stromal cell-derived factor-1 alpha (SDF-1α) were used to prepare functional small-diameter vascular grafts with enhanced anticoagulant properties [8]. However, the expensive price is not suitable for large-scale production and development. Therefore, it is necessary to create an efficient and low-cost method to improve the blood compatibility of implants [9].

An ideal medical implant should not only be conducive to the mechanical property, but also have good biocompatibility [10]. Polyester (PET) fabric, as an early developed synthetic fabric, has been used as the substrate for vascular graft because of its excellent mechanical properties [11,12,13]. It was first applied to the field of implant by military surgeons and its special structure led to the development of tissue engineering [14]. To make polyester fabrics more suitable for implantation process, many approaches has been applied to modify it, such as UV radiation [15], MW radiation [16], and US radiation [17]. However, due to the poor hydrophilicity and cell compatibility of PET fabric materials, it may lead to problems such as blood penetration and coagulation. Therefore, PET fabric scaffolds cannot meet the needs of vascular transplantation.

Therefore, to overcome the shortcomings of synthetic grafts, we consider combining synthetic fabrics with natural extracellular matrix (ECM) to give vascular grafts good mechanical and biological properties. Many studies have shown that this method of combining components with different properties plays an important role in tissue engineering, especially in vascular transplantation [18,19]. Natural ECM compositions—such as collagen, silk fibroin (SF), and gelatin (Gel)—are generally used to modify the synthetic polymer compatibility and promote cell proliferation. It has been reported that electrochemically aligned collagen filaments were used to constructing bilayer small-diameter vascular to improve endothelial cells adhesion and proliferation [20]. However, a previous study showed that collagen coating was more susceptible to perioperative vascular graft infections in vivo [21]. SF vascular graft was also coated on the knitted vascule for venous replacement in rat models. The result showed that SF had high patency and good histocompatibility, which proved that SF was a promising tissue engineering material [22]. However, due to the antigenicity of silk-sericin, degumming is a necessary process to use SF coating, which increases the complexity of vascular coating [23]. Compared with collagen and SF, the gelatin coating showed less formatted biofilms and bacterial adherence. In addition, the gelatin provides more bioactive sites to combine the vascular scaffolds with coating more tightly [24]. Therefore, in this study, we thus select gelatin to coat polyester fabric to give the PET better biocompatibility.

In addition, blood penetration has always been an important factor affecting the success of vascular transplantation. Polyurethane (PU) is considered an excellent coating material because of its excellent expansibility, biocompatibility, and processability [25,26]. Therefore, in order to overcome the problem of blood penetration, we introduced polyurethane between the PET fabric and gelatin coating [27].

**Scheme 1:**
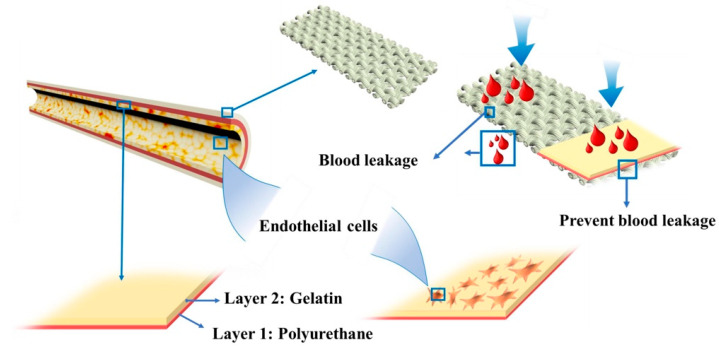
Schematic illustration showing the coating of polyurethane and gelatin on the polyester fabric and their use in preventing blood leakage and promoting endothelia cell proliferation.

The preparation of vascularized and functional coating materials using synthetic polymers and natural bioactive components is an important research subject in the field of tissue engineering. Therefore, this study used polyester textile as the substrates, gelatin coating as the natural bioactive components and polyurethane coating as impermeability components, which successfully avoid the inherent shortcomings of synthetic materials. This method provides an effective way to construct better vascular scaffolds (Scheme 1).

## 2. Materials and Methods

### 2.1. Materials

Polyester plain fabrics were bought in local chemical fiber industrial (Fa Xinrui Textile Co., Qingdao, China). Medical grade polyurethane (PU-Pellethane^®^ 2363-80AE TPU) were purchased from Dow Chemical (Guangzhou, China). Glycerol was obtained from Sinopharm Chemical Reagent (Shanghai, China). Sodium citrate anticoagulant whole blood was acquired from Beijing Bersee Science and Technology (Beijing, China). Medical grade gelatin (type A from bovine skin, ~250 bloom), glutaraldehyde (25% commercial aqueous reagent), and *N*,*N*-dimethylformamide (DMF) were supplied by Shanghai Macklin Biochemical (Shanghai, China). Human umbilical vein endothelial cells (HUVECs) were obtained from the Institute of Biochemistry and Cell Biology (Chinese Academy of Sciences, Shanghai, China). Cell culture medium and reagents were provided by Aladdin Life Technologies (Shanghai, China).

### 2.2. Coating Procedure

#### 2.2.1. PU Coating onto the Bare Polyester Fabric

PU particles (5 g) were dissolved in DMF solutions (100 mL) at 80 °C for 8 h to prepare the PU solution. The bare polyester fabric was cut into 5 × 5 cm^2^ segments, and then the PU solution was evenly gradient coated on the polyester fabric with a brush. Subsequently, the fabrics were dried in vacuum oven at 37 °C for 48 h. The fabrics developed through this process were marked as PU@PET.

#### 2.2.2. Gelatin Coating onto the Bare Polyester Fabric

Gelatin particles (4 g) and glycerol (4 g) was dissolved in deionized water (50 mL) and stirred at 37 °C for 1 h to prepare the mixture solution. The bare polyester fabric was cut into 5 × 5 cm^2^ segments, and the above solution was evenly distributed on it with a brush. Subsequently, the fabrics were dried in oven at 37 °C for 30 min. The coating and drying processes were repeated 4 times, and then the gelatin was successfully coated onto the bare polyester fabrics. The fabrics obtained by this method were marked as Gel@PET. The dried fabrics immersed in 0.1% glutaraldehyde solution for 2 min and then dried in a vacuum oven at 37 °C for 24 h.

#### 2.2.3. PU-Gel Coating on the Bare Polyester Fabric

The preparation of PU-Gel coating fabric (PU-Gel@PET) was described in Section 2.2.1 and Section 2.2.2. The PU layer was first coated on the PET and then the gelatin layer coated on the PU layer.

### 2.3. Characterization

The morphology of the fabric was observed by scanning electron microscopy (SEM) (Phenom XL, Phenomenon World, Eindhoven, The Netherlands). The average static water contact angle of coated/uncoated fabrics was measured according to the drop method (Contact Angle Meter) using data physics OCA (Data Physics, Stuttgart, Germany). Five different positions of each fabric were measured to get the average value at room temperature. Chemical structure analysis of the coating fabric was analyzed by Fourier transform infrared spectroscopy FTIR (Nicolet 50, Thermo Scientific company, Waltham, MA, USA), with a wavenumber range of 400 and 4000 cm^−1^. The chemical element composition of the fabric was analyzed by X-ray photoelectron spectroscopy (XPS) (Axis Supra^+^, Kratos Analytical, Manchester, UK), including the full spectrum and atomic ratio of carbon, nitrogen, and oxygen on different coating samples. Thermogravimetric measurements were performed from room temperature to 600 °C at a ratio of 10 °C min^−1^ (N_2_ atmosphere) on Mettler Toledo TGA/DSC 3+ STARe System (Mettler Toledo, Zurich, Switzerland). Another thermal analysis was performed by differential scanning calorimeter (DSC) (Mettler Toledo TGA/DSC 3+ STARe System, Mettler Toledo, Zurich, Switzerland) at a ratio of 10 °C min^−1^ (N_2_ atmosphere) in the temperature range of −70 °C to 200 °C. The bursting strength was tested by a universal mechanical tester (HD026 Series, HONGDA Co., Guangzhou, China). The thimble diameter of the burst strength test was 25 mm, and the moving speed was set to 50 mm/min. The samples were made into circular with a diameter of 125 mm, and 5 replicates were made for each sample. The instrument records the maximum burst strength when the fabric was destroyed by the thimble. The bursting strength was calculated by the formula

$$\mathrm{Bursting}\;\mathrm{strength}=\frac{T_{b}}{\pi \frac{D^{2}}{4}}$$

where T_b_ is the bursting force, D is the diameter of probe.

### 2.4. In Vitro Degradation

The degradation ratio was evaluated by immersing the coating fabric in phosphate-buffered saline (PBS) solution. The coating fabrics were incubated with PBS (pH = 7.4) at 37 °C for 1 month. Finally, the fabrics were dried in vacuum oven for 24 h to keep the weight constant. The percentage of weight loss for each fabric was calculated as

$$\mathrm{Weight}\;\mathrm{loss}\left(\%\right)=\frac{w_{0}-{\;w}_{t}}{w_{0}}\;\times \;100$$

where w_0_ and w_t_ presented the dry weight of the coating fabric before and after degradation at different time intervals.

### 2.5. Blood Compatibility Assays

In order to evaluate the blood compatibility of the uncoating and coating fabrics, fresh sodium citrate anticoagulant rabbit whole blood was used for platelet adhesion test, hemolysis test, whole blood adhesion, activated partial thromboplastin time (APTT), and recalcification time test.

#### 2.5.1. Platelet Adhesion Test

Anticoagulant rabbit whole blood was centrifuged at 1200 rpm for 10 min to collect platelet-rich plasma. The samples were made into circle with diameter of 14 mm and placed in 24-well plate, then 500 μL of the above plasma was added into the 24-well plate and incubated at 37 °C for 2 h. The samples were then rinsed with PBS 3 times to remove non-adhered platelets and fix the platelets with 4% glutaraldehyde for 2 h. Finally, the samples were dehydrated with gradient ethanol (30%, 50%, 70%, 90%, 95%, and 100%, respectively). The adhesion numbers and morphologies were observed by SEM after spraying gold for 90 s.

#### 2.5.2. Hemolysis Test

In the hemolysis tests, double distilled water was used as positive control, and physiological saline was served as negative control. Diluted blood was prepared with physiological saline and whole blood in the ratio of 4:5 (*v/v*). First, the samples were rinsed with PBS 3 times, then the samples were put in a conical tube containing 10 mL physiological saline. Six replicates were prepared for each sample. These conical tubes were incubated at 37 °C for 30 min, then 200 μL diluted blood was added to each conical tube, gently shaken at 37 °C for 2 h, and centrifuged at 3000 rpm for 10 min. The supernatant from each centrifuge tube was aspirated, and transferred to a 96-well plate, optical density value was read at a wavelength of 545 nm with a spectrophotometer (SpectraMax ABS). The hemolysis rate can be calculated according to the following equation.

$$\mathrm{HR}\%=\frac{A_{\mathrm{ts}}-A_{\mathrm{nc}}}{A_{\mathrm{Pc}}-A_{\mathrm{nc}}}\;\times \;100\%$$

where A_ts_, A_nc_, and A_pc_ mean the value of optical density at 545 nm of sample groups, negative groups, and positive groups, respectively.

#### 2.5.3. Whole Blood Adhesion Test

Samples were placed into 24-well plate and add 1 mL PBS to moisten them. The samples were then incubated in an oscillating shaker at 37 °C for 2 h, then 200 μL whole blood containing sodium citrate anticoagulant was added into the 24-well plate, and incubation continued for 1 h. Afterwards, the 24-well plate was taken out and gently rinsed with PBS 3 times. We then added 1 mL of 2.5% glutaraldehyde to each well to fix the whole blood for 5 min, and then placed the whole blood in refrigerator at 4 °C for 12 h. Subsequently, the samples were dehydrated with gradient ethanol (30%, 50%, 70%, 90%, 95%, and 100%, respectively). The samples were pre-frozen at −20 °C for 48 h, and then freeze-dried for 3 days. Finally, the adhesion morphologies were observed by SEM after spraying gold for 90 s.

#### 2.5.4. Activated Partial Thromboplastin Time

The samples were made into circles with a diameter of 14 mm and put into 24-well plates. Three replicates were performed for each sample group, and the blank group was used as the positive control group. Whole blood was centrifuged at 3000 rpm for 10 min to separate plasma. Then added 100 μL plasma and 100 μL APTT reagent into 24-well plate and incubated for 5 min, at which point 100 μL CaCl_2_ was added and timing was started. The 24-well plates were shaken gently to observe the liquid fluid condition. When the liquid stopped flowing, the stopwatch was stopped and the time recorded.

#### 2.5.5. Re-Calcification Time Test

The samples were made into circle with a diameter of 14 mm and put into 24-well plate. Three replicates were performed for each sample group, and the blank group was used as the positive control group. The rabbit whole blood containing sodium citrate anticoagulant was centrifuged at 1000 rpm for 10 min, and then the slight yellow suspension was drawn and labeled as PPP (platelet-deficient plasma). We added 1 mL of PPP and CaCl_2_ (0.025 mol/L) to the 24-well plates, respectively, and started timing. The 24-well plates were observed carefully. When the fibrous material began to appear, the stopwatch was stopped and the time recorded.

### 2.6. Cell Compatibility Assays

The HUVECs were cultured at 37 °C in Dulbecco’s modified Eagle medium (DMEM) containing 10% fetal bovine serum. The culture medium was replaced every two days. When the density of HUVECs reached 80%, they were digested for subsequent inoculation. All samples were made into circle with diameter of 14 mm, and 3 replicates for each sample to obtain the average value. Samples were placed in 24-well plates and fixed with a stainless steel ring. The samples were sterilized overnight at room temperature with 75% ethanol steam, and then washed completely with PBS. Samples were soaked in DMEM medium for 2 h to ensure that HUVECs could be evenly seeded. After that, the digested HUVECs were inoculated on the material at a planting density of 104 cells/well, and the medium was replaced every two days.

SEM were used to investigate the cell morphologies after 3 days of culture. First, the sample was washed with PBS, and then the cells were fixed on the sample with paraformaldehyde solution (4% concentration) at 4 °C (overnight). On the second day, the samples were dehydrated with gradient ethanol (30%, 50%, 70%, 90%, 95%, and 100%, respectively) for 20 min each time. Before imaging, the samples were dried in air for 2 h and sprayed with gold for 80 s.

The cell counting kit-8 (CCK-8) was used to study the proliferation of HUVECs on the coating samples for 1, 3, and 5 days. First, the medium was removed from the 24-well plate and the samples were rinsed with PBS, at which point 100 μL CCK-8 solution (10 μL CCK-8 + 90 μL culture medium) was added to the well. After incubating at 37 °C for 3 h, the aliquot was transferred into a 96-well plate, and the absorbance of each well was read with an enzyme labeling instrument at a wavelength of 450 nm. In this study, tissue culture plate (TCP) wells were used as control.

HUVECs cultured on samples for 3 days were used for immunofluorescence staining. The nuclei and cytoskeletons were stained with 40,60-diamidino-2-phenylindole hydrochloride (DAPI, Invitrogen, Shanghai, China) and rhodamine-conjugated phalloidin (Invitrogen, Shanghai, China), respectively, and then the cells were observed using confocal laser-scanning microscopy (Nikon, Ti2-U, Tokyo, Japan).

### 2.7. Statistical Analysis

The results were analyzed using one-way ANOVA followed by least square difference test (LSD), where *p* < 0.05 was considered statistically significant. Experimental results were presented as mean ± SD.

## 3. Results and Discussion

### 3.1. Morphological Analysis

**Figure 1:**
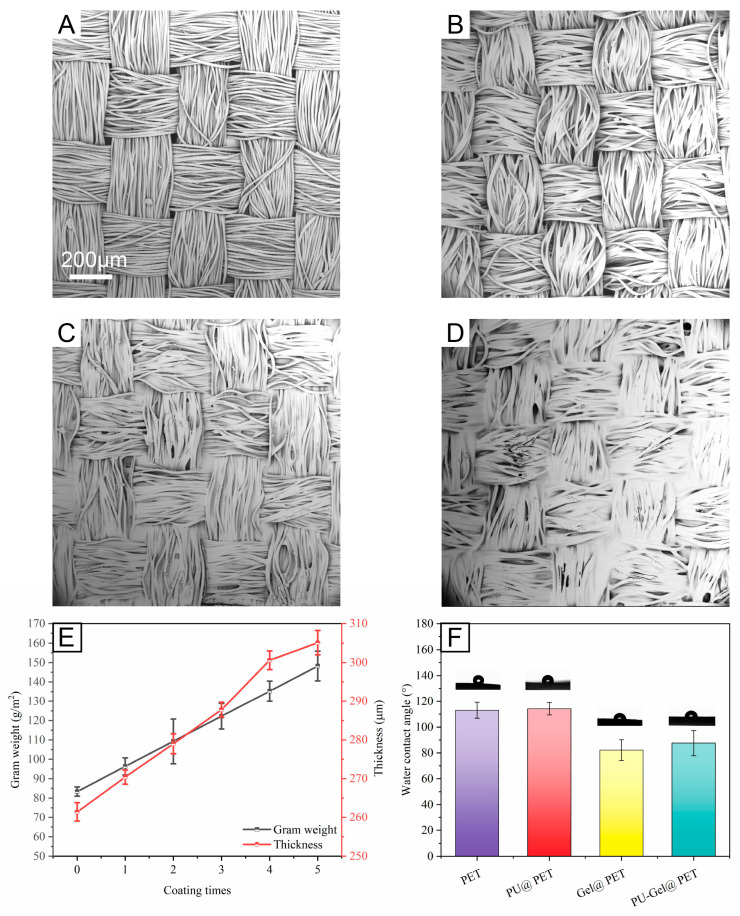
SEM images of PET and different coating fabrics (**A**–**D**). (**A**) PET. (**B**) PU@PET group. (**C**) Gel@PET group. (**D**) PU-Gel@PET group. (**E**) Relationship between weight change of coating fabric and coating times. (**F**) Static water contact angle of PET and different coatings fabrics.

According to the vascular function, the first coating should meet the requirement of preventing blood infiltration. Therefore, the polyurethane was coated on the polyester fabrics. Meanwhile, it also needs to satisfy the requirements of promoting endothelial cell migration and regeneration. Therefore, there were gelatin coatings above the polyurethane coating layer. The thickness of the material constructed by polyurethane and gelatin coating process was almost 300 μm, which was approximately close to the thickness of a human artery wall. There was a positive correlation between wall thickness and diameter. When the diameter is less than 6 mm, the wall thickness is about 400~700 μm [28]. As shown in Figure 1A–D, the SEM morphologies of polyurethane coating and gelatin coating were uniformly spread on the bare polyester, and the thickness of the coating was not as thick as that of PU-Gel@PET group. Through the numerous attempts, four-layer gelatin coating had the best effect in obatining suitable thickness and density in compliance with a human arterial vessel (Figure 1E). We also observed the coating surface of artificial vascular constructed by Terumo company, which is famous for preparation of vascular grafts and vascular equipment. The materials surface is also dense and covered the original material. In this study, we found that mixing the gelatin solution with glycerol solution at a mass ratio of 1:1 can improve the softness of the coating without damaging the cell compatibility of gelatin. Furthermore, the mechanical properties can be successfully maintained to support the blood flow.

With the increase of coating times, the surface morphologies of samples became denser, and the coating nearly covers each hole of the fabric. The internal pores of PET were filled with coating materials. This hybrid coating provided a suitable structure, with large pores in the outer layer and small pores in the inner layer. The weight of coating fabric per square meter was growing linearly with the increase of the coating times. The static water contact angles of polyester fabric with different coatings were shown in Figure 1F. The sample coating with gelatin had a lower angle, which exhibited greater hydrophilicity than bare polyester and PU coating polyester. Therefore, the gelatin coating could effectively improve the hydrophilicity of PET, thereby improving cell compatibility and reducing protein adsorption [29]. The surface of the material had a high water absorption rate, which reduced the chance of protein adhesion. The improved hydrophilicity results in biodegradation rate will be discussed in the next part [30].

### 3.2. In Vitro Degradation

**Figure 2:**
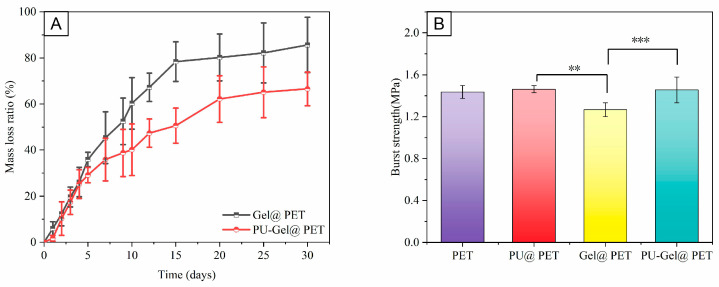
(**A**) Degradation of Gel coating and PU-Gel coating samples. (**B**) Burst strength of PET and different coating samples. (**, and *** represented *p* < 0.01, and *p* < 0.001, respectively, indicating significant dif-ferences between the two groups).

Gelatin is the product of collagen hydrolysis and is a kind of water-soluble natural polymer [31]. Therefore, once the gelatin coating material was immersed in the aqueous solution, the gelatin would biodegrade over time. The polyurethane layer would also degrade in the aqueous solutions. The ratio of degradability of coating layer was shown in Figure 2A, it can be found that of the Gel coating remains at approximately 82.2%, and that of PU-Gel coating remains at approximately 65.1% after 30 days. This mass difference of two coating layers could be conducive to adhesive force of polyurethane. One of the shortcomings of gelatin is fast degradation. The deposition of oxidized alginate and gelatin gel on PET grafts also demonstrates this characteristic, in which the gelatin coating degraded by 90% after 14 days [32].

### 3.3. Fabric Strength

It is known that polyester fabrics had excellent mechanical property. With the addition of polyurethane coating, the tensile strength increased dramatically, which could be attributed to the dense intensity of polyurethane [33]. As shown in Figure 2B, the tensile strength of PET, PU@PET, Gel@PET, and PU-Gel@PET were 1.32 MPa, 1.45 MPa, 1.26 MPa, and 1.52 MPa, respectively. This parameter is about 9 times that of Xing’s work [34], whose burst tensile is approximately 1000 mmHg, while the burst tensile of swine blood vessels is between 2000~4000 mmHg [32]. Therefore, the sample we prepared met the requirement. This is mainly due to the polyester textile excellent mechanical properties and this feature also extends the range of application. The gelatin coating samples were significantly reduced compared with pure PET samples and other samples (*p* < 0.01) which may be attributed to the brittleness of gelatin coating. The lower content of elastic gelatin can withstand force and deformation, which made the coating more rigid [35]. Thus, this coating exhibited lowest burst strength. Even though, ultimate tensile strength of PU@PET and PU-Gel@PET both showed a good agreement with the healthy arteries which was 1.5 ± 0.5 MPa due to the soft segments of polyurethane [36]. In vascular tissue engineering, the proximity (related to mechanical property) of the grafts to the natural vascule was an important factor affecting the patency of the graft. From the results of tensile strength, the addition of a polyurethane layer has thus been proved to be necessary.

### 3.4. Characterization

#### 3.4.1. Fourier Transform Infrared Spectroscopy Analysis

**Figure 3:**
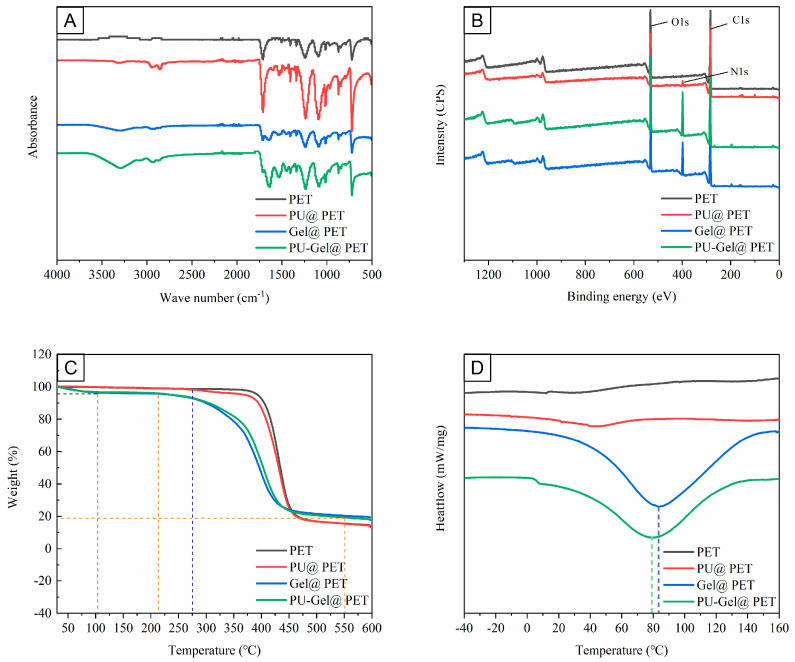
Spectrogram of PET and different coating samples. (**A**) Fourier transform infrared spectroscopy analysis. (**B**) X−ray photoelectron spectroscopy analysis. (**C**) Thermo gravimetric analysis. (**D**) Differential scanning calorimetery analysis.

Fourier transform infrared spectroscopy (FTIR) was used to characterize the gelatin and polyurethane coating on the pristine polyester. As shown in Figure 3A, there was no major difference between the pristine polyester fabrics with gelatin and polyurethane coating fabrics. Compared with the bare polyester, the polyurethane coating sample showed two new characteristic peaks at 2940 cm^−1^ and 2860 cm^−1^, which was attributed to the stretch vibration of C-H groups [31,37,38]. In addition, the two absorption peaks showed as well. Moreover, another new characteristic peak in 1540 cm^−1^ is amide Ⅱ band of urethane, in which the weak acidity of -NH group is favorable to hydrogen bonding between the substrates and polyurethane, or the polyurethane intermolecular chains [39]. The characteristic peaks in 1240 cm^−1^ and 1090 cm^−1^, are the stretching vibration of C-O groups from carbonyl group. The intensity of their peaks has increased significantly, which also indicated that the polyurethane was successfully coated. Gelatin and polyurethane coating fabrics showed the characteristic peaks at 2940 cm^−1^ and 3310 cm^−1^ due to the stretch vibration of C-H and -OH groups, respectively [40]. In the fingerprint region, new characteristic peaks appeared at 1650 cm^−1^ and 1540 cm^−1^ in the spectrogram of gelatin coating fabric, which was attributed to the stretch vibration of amide bond and bending vibrations of amide double bond [41,42]. Compared with the spectroscopy of gelatin coating, the PU-Gel coating fabric spectroscopy showed stronger characteristic peaks at 1650 cm^−1^ and 1540 cm^−1^, which was attributed to the mixture effect of polyurethane and gelatin [43]. The above results indicated that the gelatin and polyurethane were successfully coated on the pure polyester fabrics.

#### 3.4.2. X-ray Photoelectron Spectroscopy Analysis

The XPS analysis was used to further confirm the polyurethane and gelatin had been successfully coated on the fabric. The pure polyester fabric contains C and O elements, while the coating fabrics exhibited three peaks at the binding energies of 535, 404, and 284 eV, which were attributed to O, N, and C elements, respectively. This was due to the introduction of N element when the pure polyester fabric was coated [11,44,45]. The XPS survey spectra spectrum of before and after coating fabrics were shown in Figure 3B. The appearance of the N element was shown in Table 1.

#### 3.4.3. Thermo Gravimetric Analysis

Figure 3C showed the thermo gravimetric analysis (TGA) of the fabrics before and after coating. The weight of the gelatin coating fabric began to decrease slightly at 80 °C, and then in the range of 250 °C to 500 °C, the total weight loss was 80%. For uncoating fabrics, the weight started to decrease at 380 °C, which contributed to the decomposition of polyethylene terephthalate. Compared with the bare polyester fabrics, the PU coating fabrics began to decompose at 300 °C due to the polyurethane was more easily to decompose than polyester. In consideration of the above results, polyurethane and gelatin were successfully coated on the polyester fabric.

#### 3.4.4. Differential Scanning Calorimeter Analysis

Figure 3D exhibited the endothermic and exothermic conditions of PET and different coating samples. There was no significant difference between the DSC curve of PET and PU@PET. The endothermic peak usually represented the glass transition or melting behavior. In this study, since the gelatin structure was not conducive to crystallization, the endothermic peak represented the glass transition behavior. As shown in Figure 3D, there was an obviously endothermic peak at approximately 80 °C. The temperature of Gel@PET was slightly higher than PU-Gel@PET which indicated the variety of glass transition temperature. The temperature difference was caused by the introduction of PU, which made the chain segment more flexible and easier to move. In conclusion, the DSC curve also showed the gelatin and PU layer were successfully coated on the polyester.

### 3.5. Blood Compatibility Assays

#### 3.5.1. Platelet Adhesion

**Figure 4:**
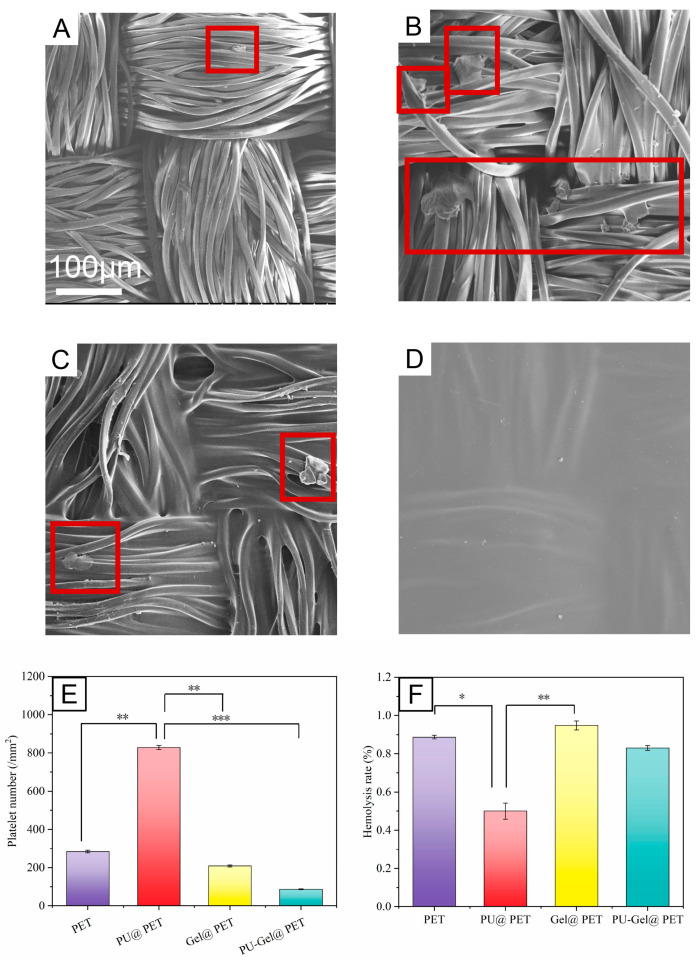
The platelet adhesion and hemolysis test of PET and different coating samples (platelets were in red boxes in **A**–**C**). (**A**–**D**) the SEM morphology of the PET, PU@PET, Gel@PET, and PU-Gel@PET samples, respectively. (**E**) The statistic number of platelet adhesion. (**F**) The hemolysis ratio of different coating samples. (*, **, and *** represented *p* < 0.05, *p* < 0.01, and *p* < 0.001, respectively, indicating significant differences between the two groups).

The degree of platelet adhesion of fabric was determined by measuring the quantity of adhered platelets. Activated platelets can not only activate a variety of coagulation factors, but their adhesion to blood contact materials is the key to cause vascular graft coagulation and thrombosis [5]. Compared with other blood cells, platelets were more sensitive to the contact of biomaterial surfaces. As shown in Figure 4E, the PU@PET group displayed a significantly higher platelet adhesion compared with PET, Gel@PET, and PU-Gel@PET groups, which indicated that the PU coating had a poor blood compatibility. There was no significant difference in platelet adhesion between the Gel@PET and PU-Gel@PET groups, thus indicating that Gel and PU-Gel coatings had great potential to inhibit platelet adhesion. In addition, the shape of platelets was approximately circular without pseudopodia, which indicated the platelets had not been activated [46]. The gelatin coating material showed obvious hydrophilicity compared with the polyurethane coating material, which may be the reason for improving the blood compatibility. From the SEM images, the PET and PU-Gel@PET group showed less platelet adhesion than other groups, which made the PU-Gel coating a potential coating technology for vascular applications.

As shown in Figure 4E, the adhesion of platelets in the four samples is between 100 and 800 per square millimeter, and the result of polyurethane coating is the highest. The results were relatively low compared with Chernonosova’s work [47]. They used a mixture of polyurethane and gelatin to prepare vascular grafts by electrospinning, and the lowest platelet adhesion was more than 1000/mm^2^.

#### 3.5.2. In Vitro Hemolysis Ratio

Hemolysis was a very simple and reliable qualitative and quantitative detection method for evaluating blood compatibility [48]. The interaction intensity between materials and erythrocytes was mainly displayed by measuring the degree of erythrocyte lysis and hemoglobin dissociation. The higher the hemolysis rate, the greater the damage to erythrocytes. In this study, only the supernatant of the positive control was red, which indicated that the erythrocytes were destroyed. However, the supernatant of the other groups was transparent, indicating that the erythrocytes were still retained as before. Figure 4F showed the specific values of different coating materials. The values of PET, PU@PET, Gel@PET, and PU-Gel@PET groups were 0.88%, 0.50%, 0.95%, and 0.83%, respectively. According to the ASTM-F 756-00 standard, the hemolysis ratio of materials in contact with blood could be classified to three categories according to the hemolysis index and its percentage. The first type of hemolysis rate is greater than 5%, which indicates the hemolysis is serious. The second type of hemolysis rate is 2–5%, which indicates mild to moderate hemolysis. The third type of hemolysis rate is less than 2%, indicating minor hemolysis, and this kind of material can be used as a non-hemolytic biological material [49]. Therefore, from the perspective of hemolysis rate, both the coated and uncoated fabrics had good blood compatibility.

As shown in Figure 4F, the hemolysis ratio is less than 1%, showing excellent anti-hemolysis. The result is also lower than Chernonosova’s work, whose hemolysis rates were between 2% and 4% [47]. Javanmard’s work reported that the hemolysis ratio of scaffold is about 2%, and that of ePTFE and is at around 4% [50].

#### 3.5.3. Whole Blood Adhesion

**Figure 5:**
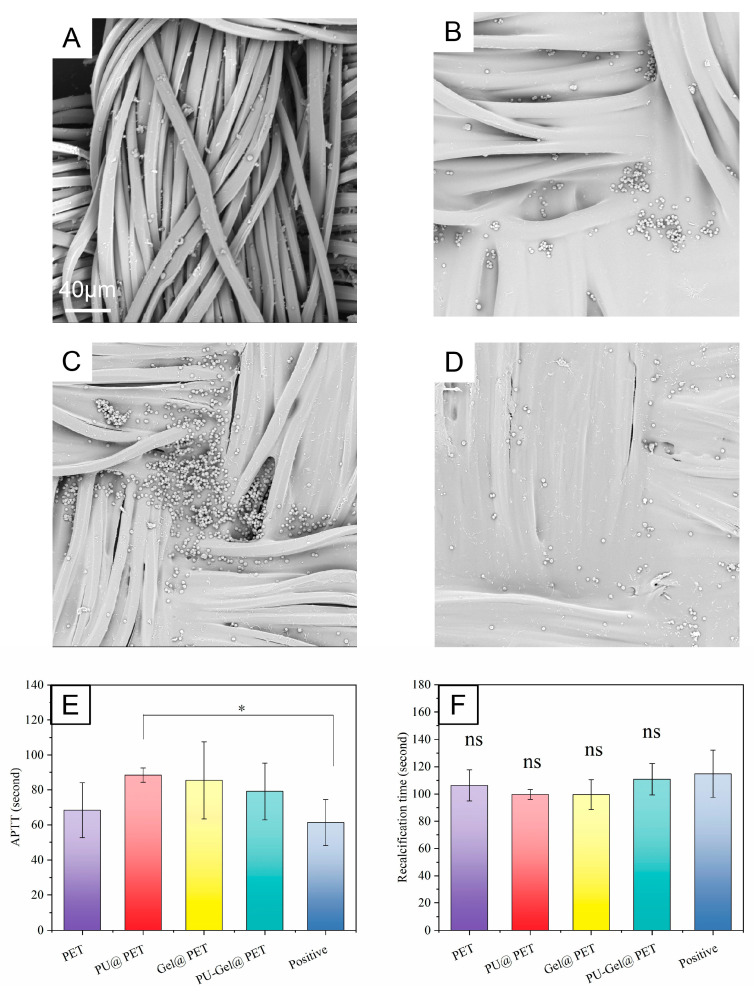
SEM morphology of whole blood adhesion condition of PET and different coating samples (**A**–**D**). (**A**) PET. (**B**) PU@PET. (**C**) Gel@PET. (**D**) PU-Gel@PET. (**E**) Activated partial thromboplastin time results, (**F**) Re-calcification time results. (* represents *p* < 0.05, indicating significant difference between the two groups, and ns represented no significant differences between with the positive group).

As shown in Figure 5, the surface of Gel@PET group had the highest erythrocyte adhesion, followed by the PU@PET group. The erythrocyte on the surface of these two coatings was in an aggregation state, which means that these two coating methods were prone to thrombus. While the PET and PU-Gel@PET group displayed in Figure 5A,C showing less erythrocyte adhesion than Gel@PET and PU@PET groups, which was because the bare polyester fabric and PU-Gel coating had smooth surface. In addition, the wetting ability of surface may also affect the adhesion of erythrocytes.

#### 3.5.4. Activated Partial Thromboplastin Time Test

Activated partial thromboplastin time is a kind of screening test to measure the coagulation factor deficiency by the endogenous pathway. Adding enough activated contact factor activator and partial thromboplastin to replace platelet phospholipid, and then adding moderate Ca^2+^, could lead to endogenous coagulation. The time from the addition of Ca^2+^ to plasma coagulation is the APTT. As shown in Figure 5E, the APTT values of the PET, PU@PET, Gel@PET, PU-Gel@PET, and positive groups were 68.4 s, 88.5 s, 85.5 s, 79.1 s, and 61.3 s, respectively. There was no significant difference between the coating groups and the positive group, except the PU coating. Furthermore, the results were consistent with the results of hemolysis ratio, which indicates the polyurethane had excellent biocompatibility.

#### 3.5.5. Re-Calcification Time Test

Plasma re-calcification was a common measurement for evaluating the function of the endogenous coagulation system. Soluble fibrinogen is converted into insoluble fibrinogen by adding Ca^2+^. Over time, insoluble fibrinogen further crosslinks to form a thrombus. The longer the time of re-calcification, the better the anticoagulation property and hemocompatibility. As shown in Figure 5F, the re-calcification time values of PET, PU@PET, Gel@PET, and PU-Gel@PET were 106.3 s, 99.8 s, 97.7 s, and 108.9 s, respectively. The glass slide served as a positive group due to its non-thrombogenic property. There was no significant difference in the values between the experimental group and the positive group. The test results showed that all the experimental groups had excellent biocompatibility.

Hemocompatibility is related to the chemical composition and hydrophilicity of the materials. The results of the contact angle showed that the gelatin coating significantly increased the hydrophilicity, which indicated that the gelatin coating was essential for improving hemocompatibility. The contact angle results were consistent with the hemocompatibility assays, in which gelatin coating had the best effect, followed by PU-Gel@PET group. Although the gelatin coating displayed the best biocompatibility, the polyurethane layer was essential for vascular application because the pure polyester and pure gelatin coating polyester fabrics could not inhibit blood leakage. Therefore, both polyurethane coating and gelatin coating are indispensable.

In summary, compared with uncoated samples, the blood compatibility of coated samples is improved, which is the result of the comprehensive action of many factors. Firstly, the hydrophilicity of the surface has changed. The addition of gelatin coating makes the surface hydrophilic, which is improves contact between material surface and blood. Secondly, the anticoagulant properties of polyurethane materials are closely related to their microphase-separated structure, which is very similar to the lining of blood vessels in living organisms. The microphase-separated structure is formed by connecting the hard and soft segments of the polyurethane structure by physical cross-linking points. Therefore, the addition of PU coating is also conducive to improve blood compatibility. Finally, the greatest credit is gelatin coating, which is similar to the substance in the inner layer of human vascular and can better restore the condition of the working blood vessels.

### 3.6. Cell Morphology and Proliferation Test

**Figure 6:**
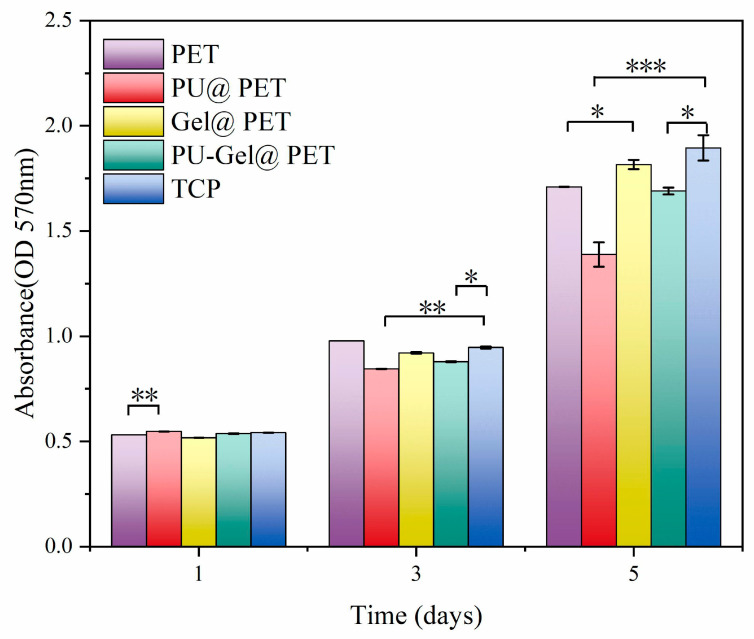
Absorbance of HUVECs on the surface of PET and different coating samples on day 1, 3, and 5. (*, **, and *** represented *p* < 0.05, *p* < 0.01, and *p* < 0.001, respectively, indicating significant differences between the two groups).

In this study, CCK-8 assay was performed to evaluate the cell proliferation on day 1, 3, and 5 after culture. The quantitative analysis of cell proliferation on different groups was shown in Figure 6. On day 1, the PU@PET group displayed the highest absorbance among the five groups. As the culture continued, the absorbance of each group began to change. The absorbance of group PET, Gel@PET, and TCP increased significantly on day 3 and day 5. Finally, the absorbance of Gel@PET group was significantly higher than that of PET, PU@PET, and PU-Gel@PET groups on day 5. This result could be attributed to the addition of gelatin coating. Animal gelatin was considered to have excellent biocompatibility due to the abundant amino acid, such as arginine-glycine-aspartate, which could regulate the cellular response and behavior and improve the biocompatibility [51]. The results showed that the cell proliferation of PU-Gel coating was better than that of pure PU coating, which can meet the proliferation requirements. At the same time, PU-Gel coating also had excellent mechanical properties and biocompatibility, which can meet the needs of artificial blood vessel transplantation.

**Figure 7:**
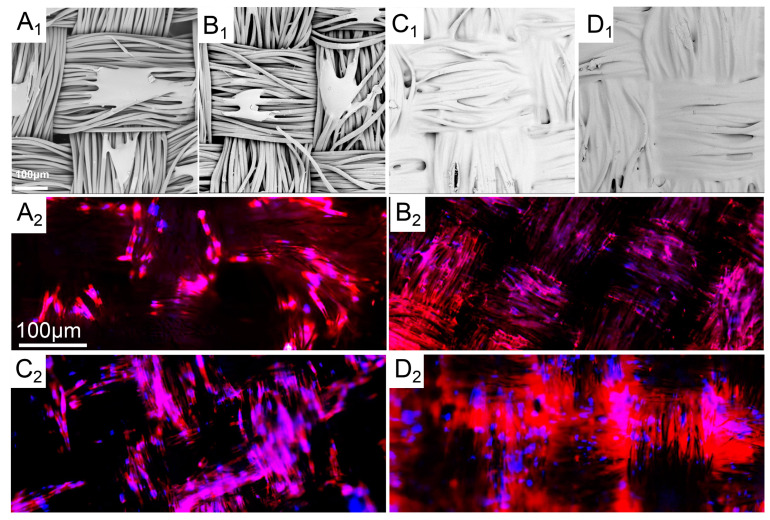
SEM and immunofluorescence staining photographs of uncoating and coating samples. (**A**_1_–**D**_1_): the SEM morphology, (**A**_2_–**D**_2_): the confocal fluorescence microscope morphology, (**A**): PET, (**B**): PU@PET, (**C**): Gel@PET, (**D**): PU-Gel@PET.

In addition to detecting the proliferation of HUVECs, we also analyzed the morphology of HUVECs by SEM and fluorescence analysis. As shown in Figure 7, the lengths of HUVECs are approximately 30–50 μm, the width and thickness are 10–30 μm and 0.1–10 μm, respectively [52]. The shape of HUVECs will change with the shape of the culture medium. In this study, HUVECs were cultured on the coating polyester fabrics. Therefore, the shape of HUVECs was consistent with the elongation of fabrics. As shown in Figure 7A_1_–D_1_, HUVECs were observed to grow well in PET and PU@PET samples, while the Gel@PET and PU-Gel@PET groups were difficult to distinguish the shape of HUVECs which was attributed to the dense coating layer merged with HUVECs maturation layer. Figure 7A_2_–D_2_ display the morphology of the stained monolayer (HUVECs) after 3 days of culturing. As expected, the PET group showed good intercellular interaction, and the gelatin coating significantly promoted the recruitment and migration of HUVECs (Figure 7A_2_,C_2_). While in the PU@PET group, the growth of HUVECs was not as ideal as the other groups, which was consistent with the test results of CCK-8 test, which may be due to the inhibitory effect of DMF on cell growth. In general, the PU-Gel@PET group showed good HUVEC morphology which demonstrated that PU-Gel bilayer on the polyester fabric was a suitable candidate for vascular coating or other implantable materials.

In summary, the mechanism of improvement in endothelial cell proliferation is similar to that in hemocompatibility. However, due to the more demanding proliferative environment for endothelial cells, the addition of PU coating does not promote the proliferation due to the introduction of organic solvents. The main effect is still from the gelatin coating.

## 4. Conclusions

In summary, we successfully coated polyurethane and gelatin on a polyester substrate for vascular applications. The polyurethane layer can effectively inhibit the blood infiltration and the gelatin layer can reduce the platelet adhesion and homolysis, and improve blood compatibility and tissue compatibility, thus promoting the infiltration and regeneration of HUVECs. This strategy has no negative effects on the tensile strength, degradation ratio, blood compatibility, and cell adhesion and migration of the coating. Considering the excellent blood compatibility and histocompatibility of this coating method, it can be applied to vascular grafts. This double-coated blood vessel, which simultaneously utilizes the mechanical properties of synthetic polymers and the degradability of natural polymers, provides a new idea for preparing vascular grafts. The vascule constructed by this method needs further animal experiments to evaluate its response in vivo.

## Figures and Tables

**Table 1:** Element content of different coating samples.

Sample	Atomic Percentage of Element (%)		
	C	O	N
PET	82.44	17.56	/
PU@PET	79.13	18.21	2.66
Gel@PET	68.15	17.58	14.27
PU-Gel@PET	65.33	18.73	15.94

## Data Availability

All data produced in this study are presented in this paper.

## References

[1] Leal B.B.J., Wakabayashi N., Oyama K., Kamiya H., Braghirolli D.I., Pranke P. (2021). Vascular Tissue Engineering: Polymers and Methodologies for Small Caliber Vascular Grafts. Front. Cardiovasc. Med..

[2] Haag S.L., Bernards M. (2020). Enhanced Biocompatibility of Polyampholyte Hydrogels. Langmuir.

[3] Cheng Y., Yang Q., Lu Y., Yong J., Fang Y., Hou X., Chen F. (2020). A femtosecond Bessel laser for preparing a nontoxic slippery liquid-infused porous surface (SLIPS) for improving the hemocompatibility of NiTi alloys. Biomater. Sci..

[4] Yi B., Yu L., Tang H., Wang W., Liu W., Zhang Y. (2021). Lysine-doped polydopamine coating enhances antithrombogenicity and endothelialization of an electrospun aligned fibrous vascular graft. Appl. Mater. Today.

[5] Lou C.W., Lu P.C., Hu J.J., Lin J.H. (2016). Effects of yarn types and fabric types on the compliance and bursting strength of vascular grafts. J. Mech. Behav. Biomed..

[6] Nerem R.M., Seliktar D. (2001). Vascular Tissue Engineering. Annu. Rev. Biomed. Eng..

[7] Niu G., Sapoznik E., Soker S. (2014). Bioengineered blood vessels. Expert Opin. Biol. Ther..

[8] Liu B., Xu F., Guo M.-Y., Chen S.-F., Wang J., Zhang B. (2013). Electrospun PLLA fibers coated with chitosan/heparin for scaffold of vascular tissue engineering. Surf. Coat. Technol..

[9] Wang W., Liu D., Li D., Du H., Zhang J., You Z., Li M., He C. (2019). Nanofibrous vascular scaffold prepared from miscible polymer blend with heparin/stromal cell-derived factor-1 alpha for enhancing anticoagulation and endothelialization. Colloids Surf. B Biointerfaces.

[10] Ding Y., Yang Z., Bi C.W.C., Yang M., Zhang J., Xu S.L., Lu X., Huang N., Huang P., Leng Y. (2014). Modulation of protein adsorption, vascular cell selectivity and platelet adhesion by mussel-inspired surface functionalization. J. Mater. Chem. B.

[11] Ma Z., Kotaki M., Yong T., He W., Ramakrishna S. (2005). Surface engineering of electrospun polyethylene terephthalate (PET) nanofibers towards development of a new material for blood vessel engineering. Biomaterials.

[12] Xu W., Zhou F., Ouyang C., Ye W., Yao M., Xu B. (2010). Mechanical properties of small-diameter polyurethane vascular grafts reinforced by weft-knitted tubular fabric. J. Biomed. Mater. Res. Part A.

[13] Tautenhahn J., Meyer F., Buerger T., Schmidt U., Lippert H., Koenig W., Koenig B. (2010). Interactions of neutrophils with silver-coated vascular polyester grafts. Langenbeck’s Arch. Surg..

[14] Riepe G., Loos J., Imig H., Schröder A., Schneider E., Petermann J., Rogge A., Ludwig M., Schenke A., Nassutt R. (1997). Long-term in vivo alterations of polyester vascular grafts in humans. Eur. J. Vasc. Endovasc. Surg..

[15] Bhatti I.A., Adeel S., Parveen S., Zuber M. (2016). Dyeing of UV irradiated cotton and polyester fabrics with multifunctional reactive and disperse dyes. J. Saudi Chem. Soc..

[16] Ghaffar A., Adeel S., Habib N., Jalal F., Haq A.-U., Munir B., Ahmad A., Jahangeer M., Jamil Q. (2019). Effects of Microwave Radiation on Cotton Dyeing with Reactive Blue 21 Dye. Pol. J. Environ. Stud..

[17] Rehman F.U., Adeel S., Saif M., Khosa M.K., Anjum M.N., Kamran M., Zuber M. (2019). Ultrasonic Assisted Improvement in Dyeing Behaviour of Polyester Fabric Using Disperse Red. Pol. J. Environ. Stud..

[18] Shi J., Chen S., Wang L., Zhang X., Gao J., Jiang L., Tang D., Zhang L., Midgley A., Kong D. (2019). Rapid endothelialization and controlled smooth muscle regeneration by electrospun heparin-loaded polycaprolactone/gelatin hybrid vascular grafts. J. Biomed. Mater. Res. Part B Appl. Biomater..

[19] Lazzeri L., Cascone M.G., Danti S., Serino L.P., Moscato S., Bernardini N. (2006). Gelatine/PLLA sponge-like scaffolds: Morphological and biological characterization. J. Mater. Sci. Mater. Electron..

[20] Zhang F., Xie Y., Celik H., Akkus O., Bernacki S., King M.W. (2019). Engineering small-caliber vascular grafts from collagen filaments and nanofibers with comparable mechanical properties to native vessels. Biofabrication.

[21] Schweizer T.A., Shambat S.M., Haunreiter V.D., Mestres C.A., Weber A., Maisano F., Zinkernagel A.S., Hasse B. (2020). Polyester Vascular Graft Material and Risk for Intracavitary Thoracic Vascular Graft Infection. Emerg. Infect. Dis..

[22] Kiritani S., Kaneko J., Ito D., Morito M., Ishizawa T., Akamatsu N., Tanaka M., Iida T., Tanaka T., Tanaka R. (2020). Silk fibroin vascular graft: A promising tissue-engineered scaffold material for abdominal venous system replacement. Sci. Rep..

[23] Haga M., Yamamoto S., Okamoto H., Hoshina K., Asakura T., Watanabe T. (2017). Histological Reactions and the In Vivo Patency Rates of Small Silk Vascular Grafts in a Canine Model. Ann. Vasc. Dis..

[24] Xiang P., Wang S.-S., He M., Han Y.-H., Zhou Z.-H., Chen D.-L., Li M., Ma L.Q. (2018). The in vitro and in vivo biocompatibility evaluation of electrospun recombinant spider silk protein/PCL/gelatin for small caliber vascular tissue engineering scaffolds. Colloids Surfaces B Biointerfaces.

[25] Zhang B., Xu Y., Ma S., Wang L., Liu C., Xu W., Shi J., Qiao W., Yang H. (2021). Small-diameter polyurethane vascular graft with high strength and excellent compliance. J. Mech. Behav. Biomed. Mater..

[26] Yang H., Zhu G., Zhang Z., Wang Z., Fang J., Xu W. (2012). Influence of weft-knitted tubular fabric on radial mechanical property of coaxial three-layer small-diameter vascular graft. J. Biomed. Mater. Res. Part B Appl. Biomater..

[27] Hong Y., Ye S.-H., Pelinescu A.L., Wagner W.R. (2012). Synthesis, Characterization, and Paclitaxel Release from a Biodegradable, Elastomeric, Poly(ester urethane)urea Bearing Phosphorylcholine Groups for Reduced Thrombogenicity. Biomacromolecules.

[28] Pellegata A.F., Asnaghi M.A., Stefani I., Maestroni A., Maestroni S., Dominioni T., Zonta S., Zerbini G., Mantero S. (2013). Detergent-Enzymatic Decellularization of Swine Blood Vessels: Insight on Mechanical Properties for Vascular Tissue Engineering. BioMed. Res. Int..

[29] Yildirim E.D., Besunder R., Pappas D., Allen F., Güçeri S., Sun W. (2010). Accelerated differentiation of osteoblast cells on polycaprolactone scaffolds driven by a combined effect of protein coating and plasma modification. Biofabrication.

[30] Mokhtari N., Kharazi A.Z. (2021). Blood compatibility and cell response improvement of poly glycerol sebacate/poly lactic acid scaffold for vascular graft applications. J. Biomed. Mater. Res. Part A.

[31] Hasan A., Memic A., Annabi N., Hossain M., Paul A., Dokmeci M.R., Dehghani F., Khademhosseini A. (2014). Electrospun scaffolds for tissue engineering of vascular grafts. Acta Biomater..

[32] Manju S., Muraleedharan C.V., Rajeev A., Jayakrishnan A., Joseph R. (2011). Evaluation of alginate dialdehyde cross-linked gelatin hydrogel as a biodegradable sealant for polyester vascular graft. J. Biomed. Mater. Res. Part B Appl. Biomater..

[33] Wu P., Nakamura N., Morita H., Nam K., Fujisato T., Kimura T., Kishida A. (2019). A hybrid small-diameter tube fabricated from decellularized aortic intima-media and electrospun fiber for artificial small-diameter blood vessel. J. Biomed. Mater. Res. Part A.

[34] Xing Y., Gu Y., Guo L., Guo J., Xu Z., Xiao Y., Fang Z., Wang C., Feng Z.-G., Wang Z. (2021). Gelatin coating promotes in situ endothelialization of electrospun polycaprolactone vascular grafts. J. Biomater. Sci. Polym. Ed..

[35] Xie X., Guidoin R., Nutley M., Zhang Z. (2010). Fluoropassivation and gelatin sealing of polyester arterial prostheses to skip preclotting and constrain the chronic inflammatory response. J. Biomed. Mater. Res. Part B Appl. Biomater..

[36] Sundback C.A., Shyu J.Y., Wang Y., Faquin W.C., Langer R.S., Vacanti J.P., Hadlock T.A. (2005). Biocompatibility analysis of poly(glycerol sebacate) as a nerve guide material. Biomaterials.

[37] Huang R., Zhang X., Li W., Shang L., Wang H., Zhao Y. (2021). Suction Cups-Inspired Adhesive Patch with Tailorable Patterns for Versatile Wound Healing. Adv. Sci..

[38] Hashim D.M., Man Y.B.C., Norakasha R., Shuhaimi M., Salmah Y., Syahariza Z.A. (2010). Potential use of Fourier transform infrared spectroscopy for differentiation of bovine and porcine gelatins. Food Chem..

[39] Xue N., Wang W., Chen Z., Heng Y., Yuan Z., Xu R., Lei C. (2022). Electrochemically stable poly (vinylidene fluoride)-polyurethane polymer gel electrolytes with polar β-phase in lithium batteries. J. Electroanal. Chem..

[40] Lin J., Pan D., Sun Y., Ou C., Wang Y., Cao J. (2019). The modification of gelatin films: Based on various cross-linking mechanism of glutaraldehyde at acidic and alkaline conditions. Food Sci. Nutr..

[41] Kong X., He Y., Zhou H., Gao P., Xu L., Han Z., Yang L., Wang M. (2021). Chondroitin Sulfate/Polycaprolactone/Gelatin Electrospun Nanofibers with Antithrombogenicity and Enhanced Endothelial Cell Affinity as a Potential Scaffold for Blood Vessel Tissue Engineering. Nanoscale Res. Lett..

[42] Wu T., Zhang J., Wang Y., Sun B., Guo X., Morsi Y., El-Hamshary H., El-Newehy M., Mo X. (2017). Development of Dynamic Liquid and Conjugated Electrospun Poly(L-lactide-co-caprolactone)/Collagen Nanoyarns for Regulating Vascular Smooth Muscle Cells Growth. J. Biomed. Nanotechnol..

[43] Nagiah N., Johnson R., Anderson R.H., Elliott W., Tan W. (2015). Highly Compliant Vascular Grafts with Gelatin-Sheathed Coaxially Structured Nanofibers. Langmuir.

[44] Wang D., Xu Y., Wang L., Wang X., Yan S., Yilmaz G., Li Q., Turng L.-S. (2020). Long-term nitric oxide release for rapid endothelialization in expanded polytetrafluoroethylene small-diameter artificial blood vessel grafts. Appl. Surf. Sci..

[45] Alizadehaghdam M., Abbasi F., Khoshfetrat A., Ghaleh H. (2013). Microstructure and characteristic properties of gelatin/chitosan scaffold prepared by a combined freeze-drying/leaching method. Mater. Sci. Eng. C.

[46] Tang G.Z., Wang Y.J., Guo G.W., Ma X.X., Wang L.Q. (2010). Effect of Micro Pits Topography on the Platelets Adhesion on 316L Stainless Steel Surface. Adv. Mater. Res..

[47] Chernonosova V.S., Gostev A.A., Chesalov Y.A., Karpenko A., Karaskov A.M., Laktionov P.P. (2018). Study of hemocompatibility and endothelial cell interaction of tecoflex-based electrospun vascular grafts. Int. J. Polym. Mater. Polym. Biomater..

[48] Gao J., Guo H., Tian S., Qiao Y., Han J., Li Y., Wang L. (2019). Preparation and mechanical performance of small-diameter PHBHHx vascular graft by electrospinning. Int. J. Polym. Mater. Polym. Biomater..

[49] Wu T., Zhang J., Wang Y., Sun B., Yin M., Bowlin G.L., Mo X. (2018). Design and Fabrication of a Biomimetic Vascular Scaffold Promoting in Situ Endothelialization and Tunica Media Regeneration. ACS Appl. Bio Mater..

[50] Javanmard S.H., Anari J., Kharazi A.Z., Vatankhah E. (2016). In vitro hemocompatibility and cytocompatibility of a three-layered vascular scaffold fabricated by sequential electrospinning of PCL, collagen, and PLLA nanofibers. J. Biomater. Appl..

[51] Su K., Wang C. (2015). Recent advances in the use of gelatin in biomedical research. Biotechnol. Lett..

[52] Krüger-Genge A., Blocki A., Franke R.-P., Jung F. (2019). Vascular Endothelial Cell Biology: An Update. Int. J. Mol. Sci..

